# Pathomechanisms of Prenatally Programmed Adult Diseases

**DOI:** 10.3390/antiox12071354

**Published:** 2023-06-28

**Authors:** Endre Sulyok, Balint Farkas, Jozsef Bodis

**Affiliations:** 1 National Laboratory on Human Reproduction, University of Pécs, 7624 Pécs, Hungary; esulyok@t-online.hu (E.S.); bodis.jozsef@pte.hu (J.B.); 2 Faculty of Health Sciences, Doctoral School of Health Sciences, University of Pécs, 7624 Pécs, Hungary; 3 MTA–PTE Human Reproduction Scientific Research Group, 7624 Pécs, Hungary; 4 Department of Obstetrics and Gynecology, School of Medicine, University of Pécs, 7624 Pécs, Hungary

**Keywords:** perinatal programming, epigenetic modifications, oxidative stress, placental function, nutrition, metabolic hormones

## Abstract

Based on epidemiological observations Barker et al. put forward the hypothesis/concept that an adverse intrauterine environment (involving an insufficient nutrient supply, chronic hypoxia, stress, and toxic substances) is an important risk factor for the development of chronic diseases later in life. The fetus responds to the unfavorable environment with adaptive reactions, which ensure survival in the short run, but at the expense of initiating pathological processes leading to adult diseases. In this review, the major mechanisms (including telomere dysfunction, epigenetic modifications, and cardiovascular–renal–endocrine–metabolic reactions) will be outlined, with a particular emphasis on the role of oxidative stress in the fetal origin of adult diseases.

## 1. Introduction

The concept of developmental origin of certain chronic diseases later in life was established by Barker and co-workers. It was based on observations in that increased coronary heart disease mortality rates were inversely related with the birthweight in a cohort of patients in Hertfordshire, United Kingdom. According to this concept, adverse events at a critical period of development induce silent pathophysiological alterations that may progress to long-lasting consequences in adulthood [1,2,3,4,5].

The association between low birthweight with hypertension, type 2 diabetes, and other components of metabolic syndrome has been confirmed in both human epidemiologic studies and in experiments of animals from various species. Classically, it has been claimed that undernourished fetuses adapt to a limited placental nutrient supply to ensure survival in the short run, but at the expense of durable complex reactions that may lead to unfavorable long-term consequences. In support of this notion, the “thrifty phenotype hypothesis” was postulated claiming that the adaptive response to adverse fetal environment protects key organs, but at the cost of non-vital organ systems [6]. These complex adaptive responses are regarded as the expression of developmental plasticity which permits a single genotype to manifest as different phenotypes according to the environmental influences [7,8]. Developmental plasticity is confined to a relatively short period, “a critical window” of the prenatal and early postnatal life when environmental stimuli/insults can program the phenotypes that may manifest as certain chronic diseases later in life [9,10,11].

Although it has been clearly demonstrated that low birthweight is a risk factor for adult diseases, it also has to be considered that newborns of this group comprise premature infants with a gestational age of ≤37 weeks and small-for-gestational-age neonates with a birthweight below the 10th percentile for their gestational age. This distinction appears to be reasonable as the nature, intensity, and duration of these intrauterine insults/stresses may be markedly different [12,13]. Further analysis of the relationship between birthweight and adult diseases revealed a U-shaped pattern, indicating that large-for-gestational-age/overweight neonates are also at a possibly increased risk of adult diseases due to fetal exposure to altered metabolic/endocrine milieu as it has been observed in maternal diabetes. Accelerated growth rates (catch-up growth) of low birthweight neonates during infancy and early childhood has also been claimed to contribute to the developmental programming of cardiovascular diseases. These observations provided a basis for nutritional interventions to prevent or to reduce the risk of developmentally programmed adult diseases. Concerning maternal nutritional manipulations, three sensitive developmental windows have been defined with different long-term outcomes. These comprise the embryonic period, placental development, and fetal growth, respectively [14].

**Figure 1:**
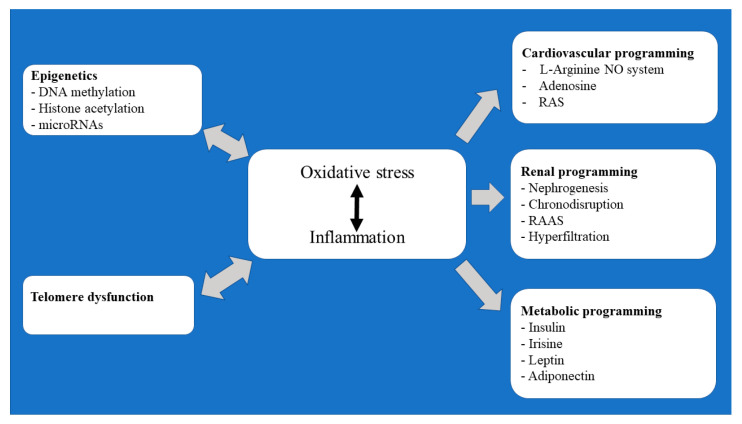
Flow chart summary of the pathomechanisms underlying the prenatally programmed adult diseases.

In this review, an attempt has been made to outline some of the major elements underlying the developmental programming of cardiovascular, renal, and metabolic/endocrine diseases. Particular attention has been made to reprogramming by epigenetic modifications and telomere dysfunction that occur during oocyte maturation and early embryonic development (Figure 1).

## 2. Telomere System and Fetal Programming

### 2.1. The Function of the Telomere/Telomerase System

Telomeres consist of non-coding, guanine-rich tandem repeats of TTAGGG and the protein complex. They serve as a cap-like structure to maintain genomic integrity and stability [15,16]. Upon each cell division telomere lengths decrease by 50–200 base pairs, and without efficiently operating their protective mechanisms this could progress to cellular senescence and apoptosis [17,18]. Telomere lengths can be maintained by the enzyme telomerase, which is abundantly expressed in stem cells, germ cells, and regenerating tissues, but not in somatic cells, and are therefore not able to compensate for the successive losses of telomere base pairs and undergo telomere shortening [19,20].

Telomerase is a ribonucleoprotein enzyme that adds new telomere sequences to the ends of chromosomes. It is composed of the catalytic unit, telomerase reverse transcriptase (TERT), and its RNA template (TERC) [21,22]. These components are associated with six individual proteins of the shelterin complex and several nuclear proteins that function to assist telomere assembly, trafficking, and stability [23,24]. Mutations or deletion of any of the components of the telomerase complex may result in telomere shortening, dysfunction, accelerated aging, and different clinical manifestations of telomeropathies [25,26,27,28,29,30].

### 2.2. Telomeres and Developmental Programming

Intensive research in the field linked telomere function to the fetal programming of health and disease risk later in life. Comprehensive reviews have provided experimental and clinical evidence that the telomere system may serve as a common underlying mechanism of several age-related disorders [30,31]. The association of telomere shortening with reproductive aging and failed assisted reproduction has also been established [32,33,34].

It has been shown that telomere length undergoes developmental regulation. It is elongated in the germ cells and early embryos and remains stable in the fetal period followed by an accelerated shortening in infancy corresponding to a rapid postnatal growth. In adults telomeres shorten further, but the rate of shortening is progressively reduced with advancing age [27,28,29,35]. Animal studies have shown that postnatal catch-up-growth programs telomere dynamics and is associated with a shorter life span and shorter telomeres [36,37].

The age-related decline in telomere length/function is dependent on their initial endowment [38] that is determined by genetic, environmental, and lifestyle factors. The importance of the initial set of telomeres was supported by the study of Moreno-Palomo et al., which demonstrated that newborns with a shorter cord blood telomere length presented significantly higher levels of basal and mitomycin-C-induced genetic damage when compared to those with longer telomeres [39]. To explore the contribution of genetic and environmental factors to the age-dependent telomere attrition a longitudinal study was conducted with mono- and dizygotic same-sex twin models. From this study, it was clearly demonstrated that the early life environment was the main determinant underlying telomere dynamics throughout the human life course; 72% vs. 28% for environmental and genetic factors, respectively [40].

Intrauterine hypoxia of various causes modulates hypoxia-inducible factor 1α (HIF-1α) expression, which directly mediates telomerase activity in the placental tissue and embryonic stem cells to maintain telomere integrity by increasing the cellular antioxidant defenses [41,42,43].

Trophoblasts in placentas from women with pregnancies complicated by intrauterine growth restriction (IUGR) exhibited a decreased activity of telomerase along with shorter telomeres, which suggested that compromised telomere functions may promote senescence and apoptosis in the placental tissue [44,45,46]. In line with these observations, a previous analysis of placental tissue from growth discordant twins revealed a significantly higher telomerase activity in the larger than in the smaller twin [47].

It has also been recognized that maternal stress during pregnancy is associated with telomere shortening, and induces endocrine, oxidative, and immune responses that can influence the fetal environment and lead to the manifestation of fetal phenotypes that increase the risk of adult diseases [16]. Particular attention was paid to explore the importance of chronic or excessive psychological stress and telomere dysfunction [48,49]. It has been shown that stress-induced endogenous cortisol or exposure to exogenous cortisol inhibit telomerase expression and result in telomere shortening [50,51,52]. Interestingly, Enlow et al. provided evidence in that hair cortisol levels in pregnant mothers appeared to have sex-specific effects on newborn telomere lengths measured in the cord blood leukocytes as cortisol levels were found to be associated with the telomere length of female but not of male neonates [53]. In addition to cortisol, estradiol and testosterone have also been shown to play a role in the establishment of fetal telomere setting by stimulating telomerase expression in luteinized granulosa cells [54,55].

The excessive generation of reactive oxygen species (ROS) in the maternal and foeto-placental compartments in various pregnancy pathologies has been regarded as a major factor causing telomere dysfunction and compromising oocyte development, fertilization, and embryo formation [56,57]. As telomeres are composed of short, guanosine-rich tandem repeat sequences that are particularly prone to oxidative damage [58], it has been relevant to claim that oxidative stress (OS) accelerates telomere shortening, decreases telomerase activity, and induces senescence/apoptosis via the DNA damage-induced activation of the p53 pathway [59].

Most recently, it has been reported that the telomerase RNA transcript can generate two dipeptide repeat proteins: the highly charged valine-arginine and the hydrophobic glycine-leucine repeats. Cells undergoing telomere dysfunction may abundantly express these dipeptide-repeat proteins with the potential to alter the cellular stress responses [60]. These observations may open new avenues to further explore the biological and clinical significance of telomeres.

## 3. Epigenetic Modifications and Developmental Programming

Epigenetic modifications mediate environmental influences of the genome and have been shown to operate during folliculogenesis, oogenesis, fertilization, and em-bryonic/fetal development. Epigenetic patterns have been claimed to contribute to fe-male reproductive aging and reproductive outcome [61,62]. By definition, epigenetics are heritable changes that affect gene expression without altering the nucleotide se-quences of DNA. It is achieved by four major mechanisms: (a) DNA methylation (b) histone acetylation, microRNAs expression and (c) nucleosome positioning [63,64].

### 3.1. DNA Methylation

DNA methylation occurs predominantly in the cytosine bases preceding guanine bases (CpG sites) and involves the covalent addition of a methyl group of S-adenosylmethionine (SAM) to the fifth carbon position of cytosine bases in the CpG sites. This reaction is mediated by DNA methyltransferases (DNMTs) and yields 5-methyl-cytosine and S-adenosylhomocysteine (SAH). Two types of methylation have been documented; maintaining methylation is catalyzed by DNMT1, while de novo methylation is performed by DNMT3a and DNMT3b, respectively [65]. DNMT methylation is involved in the regulation of gene expression; DNA hypermethylation may be associated with an inactive chromatin structure, the limited binding of transcription factors to their gene promoters, and gene silencing. In contrast, DNA hypomethylation enables transcription factors to bind to their gene promoters and activate gene transcription [66].

Methylation of the arginine residues of histone and non-histone proteins as post-translational modifications can also affect the DNA structure and function along with several cellular functions, including the cell cycle and apoptosis [67].

The methylation pattern is developmentally regulated. Methylation–demethylation cycles undergo dynamic changes to establish the totipotency of the embryo. Expression of DNMTs and DNA methylation increases in the growing oocytes until stage M II, and after fertilization active and passive genome-wide demethylation takes place in the zygote and early cleavage states. This process is followed by the global de novo methylation mostly of the inner cell mass of blastocysts [68].

DNA demethylation can be achieved through several mechanisms, including oxidative demethylation catalyzed by ten-eleven-translocation (TET) proteins. These enzyme reactions generate 5-hydroxy-methylcytosine (5-hmC) from 5-methylcytosine (5meC). As TET enzymes are differentially expressed in oocytes, zygotes, blastocysts, and preimplantation embryos, this oxidative product can be detected of various intensities in the different developmental stages. It also has to be noted that 5hmC can be oxidized further by these TET enzymes to form aldehyde and carboxylic acid, respectively [69,70].

Altered DNA methylation in germ cells or in the early embryo has the potential to compromise genomic imprinting during development and induce disease phenotypes that may manifest imminently during the early developmental stages, postnatally, or even later in adult life. In this regard, the association of DNA methylation with advanced maternal age and with assisted reproductive technologies has been widely studied. A recent mRNA-seq and genome-wide DNA methylation study revealed significant, non-random changes in the features of the transcriptome and DNA methylome in human ovarian granulosa cells as women age and their ovarian functions deteriorate. Importantly, the poorly or highly methylated DNA regions of young women were further reduced or increased, respectively, as the women aged, resulting in differentially expressed genes in the two age-groups [71].

Recently, Yang et al. evaluated the genome-wide methylation profile of human preimplantation embryos. In trophoectoderm biopsy samples obtained from blastocysts, they demonstrated negative correlations of genome-wide methylation levels to the embryo quality and maternal age, thereby confirming that increased levels of DNA methylation may compromise embryo competence [71]. It is also to be noted that variations of DNA methylation in samples from the placental and umbilical cord tissues, saliva, and cord blood of low-birthweight neonates have been claimed to mediate perinatal programming of several diseases later in life. Genome-wide methylation studies have revealed an association between differentially methylated DNA, neurodevelopmental impairments, and compromised immune functions in neonates [72,73,74,75].

From a clinical point of view, it has to be stressed that there are marked differences in the DNA methylation profiles of human oocytes and preimplantation embryos between in vitro and in vivo conceived children, which can be attributed to the assisted reproductive technologies (culture media and ovarian stimulation) rather than infertility [76,77,78,79,80].

### 3.2. Histone Acetylation

Histone acetylation has an essential role of post-translational modification. It is controlled by two groups of opposing enzymes: histone acetyltransferases (HATs) and histone deacetylases (HDACs), respectively. It has been shown that deletion of both the HDAC1 and HDAC2 genes in mouse oocytes results in a reduced ovary size, arrest of follicle development at the secondary follicle stage, perturbed oocyte transcriptome, apoptosis induction, and infertility [81]. Furthermore, animal and human studies have demonstrated that the acetylation levels of several histone lysine residues in oocytes were closely related to the maternal age. Namely, an advanced maternal age was found to be associated with defective deacetylation and higher levels of oocyte acetylation [82]. In support of these findings, HDAC inhibition with trichostatin A induced accelerated oocyte aging. In contrast, young oocytes and caffeine-treated delayed aging oocytes were found to be associated with hypoacetylation, and appeared to be protected from spindle defects, chromosome misalignments, aneuploidy, and embryo loss [83].

A quantitative analysis of acetylated proteomics in granulosa cells from patients with polycystic ovary syndrome has revealed a widespread lysine acetylation of proteins, and enhanced acetylation was found to be associated with the markedly reduced rates of two pronuclear cells and available embryos [84]. Importantly, histone acetylation on lysine residues in mouse ovarian granulosa cells was found to be stimulated by FSH [84,85], and in vitro maturation downregulated the expression of HDAC mRNA in M II oocytes and early cleavage embryos [86]. However, others did not detect these differences in the histone modification patterns in the in vivo and in vitro mouse embryos from the zygote to blastocyst stage [87].

Sirtuins (SIRTs) are highly conserved protein family of NAD+-dependent histone deacetylases that confer protection against aging and age-related disorders. In mammals seven members of the SIRT family have been identified (SIRT1-SIRT7) with different subcellular localization, tissue specificity, activity and functions [88]. In general, SIRTs regulate cellular metabolism, redox state, stress signaling, cell cycle and genome stability. With respect to female reproduction, SIRT 1, SIRT 3 and SIRT 6 are closely related to ovarian aging and related decline in ovarian reserve [88,89]. SIRT 1-deficient mouse has ovarian dysfunction and compromised oocyte developmental potential [90], SIRT3 depletion resulted in downregulation of steroidogenic enzymes [91,92], while SIRT 6 has been documented to regulate DNA repair and telomere maintenance when challenged by OS. In mouse oocytes SIRT6 depletion caused inadequate histone deacetylation, chromosome mis-segregation, aneuploidy with subsequent defects in embryonic development [93].

In addition to acetylation, histones can also be modified by methylation, phosphorylation, ubiquitination, and sumoylation. All these modifications proved to be important regulators of gene transcription; depending on the site of their action, they may promote or inhibit transcription processes [94].

It can be concluded that epigenetic modifications play a crucial role in both normal development, and in the development of the clinically relevant imprinting disorders. This is a dynamic mechanism that integrates the tightly controlled interactions of its individual elements. It can be modified by oxidative metabolism, and can be involved in basic biological processes, including telomere dysfunction, cellular senescence, and apoptosis [95,96].

In our in vitro fertilization (IVF) settings, SIRT1 and SIRT6 mRNA expressions were found to be positively related to mature oocytes and clinical pregnancy [97]. Moreover, DNMT3a mRNA levels in the follicular fluid proved to be higher in pregnant than in non-pregnant patients, and granulosa cell HDAC6 mRNA expression was found to be associated with the number of retrieved and MII oocytes, and good quality embryos [98].

### 3.3. MicroRNA

#### 3.3.1. Pathophysiology

MiRNAs are short, non-coding single-stranded RNA molecules of approximately 18–24 nucleotides (nt) in length. This functional miRNA is produced via stepwise actions of enzyme complexes to form primary miRNA, then the 70–90 nt precursor, and then pre-miRNAs that are further processed to the 18–24 nt double-stranded miRNAs. The final single-stranded mature miRNAs are generated by the elimination of one strand. This final, single-stranded miRNA binds to the 3′-untranslated regions of the target mRNA and causes either its degradation or its transcriptional repression. MiRNA-coding genes are distributed across chromosomes either individually, or in clusters thereby allowing synergistic patterns of transcription and their related regulatory functions. Importantly, one single miRNA can regulate more than one gene, and conversely, an individual gene can be regulated by multiple miRNAs [99]. Evidence has been provided in that miRNAs are involved in diverse biological processes, including proliferation, differentiation, migration, and apoptosis, and have been proven to be essential for sexual differentiation, gonadal development, gametogenesis, and reproductive performance [100,101].

#### 3.3.2. Fetal Programming

Recent studies attempting to explore the involvement of miRNA–mRNA networks in the fetal programming of adult diseases have shown differential expressions of the placental and circulating miRNAs in pregnancies complicated by preeclampsia, intrauterine growth restriction, and recurrent pregnancy loss [102,103]. Moreover, altered miRNA expression has been documented in mothers on a high-fat diet, with pre-pregnancy obesity [104], and with insulin resistance [105]. These patterns of miRNA expression are likely to be related to adipogenesis and to the later development of metabolic disorders in children born to the affected mothers. Furthermore, it has to be noted that maternal nutrient restriction can also cause permanent alterations in miRNA expression in the aortas of the newborn and aging rat offspring [106].

## 4. Oxidative Stress (OS) and Fetal Programming

### 4.1. Pathophysiology

Reactive oxygen species (ROS) are generated as a normal product of cellular metabolism and have a regulatory role in several cellular processes. When their excessive generation exceeds the capacity of antioxidant defense mechanisms, OS ensues and ROS reacts with cellular lipids, proteins, and DNA, thereby causing cellular dysfunction, damage, and apoptosis. This contention is consistent with the “quiet metabolism” concept, which claims that there are upper and lower limits of metabolic normality [107].

During pregnancy, the physiological generation of ROS occurs in association with several developmental processes, including oocyte maturation, fertilization, implantation, and embryonic/fetal development [108]. During gestation, OS may impair placental function and morphology. In the first trimester, OS induces vacuolization in syncytiotrophoblasts and reduces the villous surface area. In the second and third trimesters, it is associated with the sustained increase in the placental vascular resistance and impaired blood supply to the fetus that results in ischemia-reperfusion injury [109].

In addition to ROS production, hypoxic insults can activate inflammatory cells and enhance the production of inflammatory mediators culminating in the fetal systemic inflammatory response syndrome. Inflammation in turn leads to an increased ROS release, causing a vicious circle to ensue. Inflammatory responses to OS can be achieved via the activation of the TLR-4 mediated NF-κB and IL-6/STAT 3 signaling pathways. Hypoxia-induced IL-1β and HIF-1 may also contribute to these mechanisms [110].

Importantly, in response to hypoxia the HIF-1 pathway is activated, which mediates the generation of certain cytokines including erythropoietin (EPO). EPO is the main regulator of erythropoiesis and exhibits protective effects in various maternal and fetal tissues due to its anti-apoptotic, anti-oxidative, anti-inflammatory, cell proliferative, and angiogenic properties. Newborn infants exposed to perinatal asphyxia have elevated EPO levels that may serve as a marker of severity of hypoxic-ischemic brain injuries, and also as an adaptive response against hypoxia to mitigate tissue damage [111]. Similar protective EPO effects have been demonstrated in pregnancies complicated by pre-gestational and gestational diabetes [112]. It is of note, however, in that a recent, multicentric, double-blinded, randomized, placebo-controlled trial found no significant effects of EPO on mortality or neuro-developmental impairments in a large cohort of asphyxiated neonates [113].

### 4.2. Fetal Programming

OS and inflammation are regarded as the major pathogenetic factors in initiating and maintaining endothelial dysfunction, placental aging-related pathologies, and adverse pregnancy outcomes [114].

The most frequently encountered pregnancy pathologies include pregnancy-induced hypertension, pre-eclampsia, intrauterine growth restriction, gestational diabetes mellitus, preterm delivery, and twin pregnancy.

In addition to these obstetric complications, maternal under/overnutrition, obesity, excess gestational weight gain, and exposure to glucocorticoids, environmental pollutants, drugs, toxic agents, and several lifestyle factors (stress and physical activity) are also known to create an adverse fetal environment. It has been established that all these environmental stress factors may increase the production of ROS and pro-inflammatory cytokines and may alter the expression of the related genes. As a result, adaptive responses may occur with cellular dysfunction, compromised organ development, and programming diseases, especially cardiometabolic and renal diseases later in life [115].

## 5. Fetal Programming of Cardiovascular Diseases

The pioneer discovery by D Barker and co-workers prompted clinicians and research groups to explore the underlying mechanisms of the impact of adverse intrauterine conditions on cardiovascular health later in life. It has been widely recognized that episodic or chronic fetal hypoxia and their related OS induces fetal cardiovascular defenses to hypoxia. This defense is achieved by the redistribution of blood flow from the peripheral circulation to the vital organs due to the increase in the fetal peripheral vascular resistance. The mechanisms mediating the fetal peripheral vasoconstrictor response to hypoxia involve neural, endocrine, and local paracrine factors [116]. Nitric oxide (NO) generation by the endothelium and its bioavailability plays a pivotal role in determining the local vascular tone.

Endothelial progenitor cells are involved in the regulation of vascular health and repair. They have been claimed to maintain the structure and function of endothelial cells by repairing the damaged endothelial tissue [117]. Intrauterine growth-restricted adult offspring of Wistar rats were found to have impaired endothelial functions as indicated with their reduced vascular reactivity, decreased NO production, and increased phosphorylation of eNOS. These changes were associated with a reduced functional capacity and an accelerated senescence of endothelial progenitor cells derived from the bone marrow [118]. Vascular endothelial growth factors (VEGFs) are also implicated in the maturation and differentiation of the fetal vasculature. They influence the expression and organization of contractile proteins in the arterial smooth muscle cells and via the upregulation of their receptors (VEGF-R1 andVEGF-R2), which aid in mediating the vascular remodeling caused by chronic hypoxia [119,120]. In addition, VEGF-C has been demonstrated to stimulate endothelial NO generation that may protect the functional integrity of the developing vasculature exposed to chronic hypoxia [121].

### 5.1. L-Arginine–NO–ADMA System and Fetal Programming

#### 5.1.1. Metabolic Pathway of NO Generation and Elimination

L-arginine uptake into the cells is mediated by cationic amino acid transporters (CATs), where it serves as a substrate for NO synthase (NOS) isoforms (neuronal nNOS, endothelial eNOS, and inducible iNOS, respectively), for NO generation, and for arginase that hydrolyzes l-arginine to urea and l-ornithine. Arginase competes with NOS for their common substrate, l-arginine, meaning that it can reduce l-arginine bioavailability by redirecting l-arginine catabolism from NO to l-ornithine, which is the precursor of polyamines and proline. These substances may induce vascular smooth muscle cell growth and proliferation and collagen deposition that may progress to neointima formation and remodeling of the vascular wall [122]. Arginase can further impair NO generation by NOS uncoupling, and by the production of superoxide and peroxynitrite [123]. NOS isoforms are competitively inhibited by asymmetric dimethylarginine (ADMA) and monomethylarginine (MMA), which are formed from the methylated arginine residues of proteins by the methionine-dependent trans-methylation reaction catalyzed by protein methyltransferases (PRMTs). ADMA and MMA are mainly metabolized by the dimethylarginine dimethylaminohydrolases (DDAHs) enzymes to form dimethylamine and citrulline. Decreased expression/activity of DDAH and enhanced activity of the PRMT result in the cellular accumulation of ADMA and MMA. Elevated levels of ADMA and MMA inhibit NO production and favor superoxide formation [124]. Enzymes involved in the l-arginine metabolism are regulated in a redox-sensitive fashion; reactive oxygen species can increase the activity of PRMT, inhibit DDAH, and upregulate arginase. As a result, superoxide/peroxynitrite production increases, leading to an impaired NO generation and a compromised endothelial function [125,126]. Excessive ROS generation reduces NO bioavailability, increases the superoxide anion to NO ratio, and promotes vasoconstriction. Superoxide, therefore, is likely to play a role in the tonic regulation of the placental circulation [127].

#### 5.1.2. L-Arginine NO System and Fetal Programming

Placental tissue expresses eNOS/iNOS, their endogenous inhibitor ADMA, and the ADMA-metabolizing enzymes DDAHs. In pregnancies complicated by preeclampsia and/or intrauterine growth retardation, circulating ADMA levels are elevated, and NO generation and their related utero-placental circulation is impaired. These alterations have been proposed to be associated with a reduction in the placental size, decreased trophoblast invasion, and failure of uterine vessel remodeling [128]. Consistent with these findings, Ayling et al. reported that the overexpression of DDAHs in the first trimester human placenta and chorionic villous explant reduced ADMA production and increased NO and cGMP generation, with subsequent stimulations of trophoblast cell mobility and invasion [109]. It has been proposed that in these complex processes VEGFs are also involved to promote placentation [129,130].

Hypoxic insults and OS in early life may induce ADMA-related NO/ROS imbalance, resulting in an increased fetal vascular tone that is considered as an important key factor for programming adult hypertensive disorders [131,132]. Early signs of these processes include fetal aortic wall thickening, large artery stiffness, and reduced distensibility [133,134,135,136].

### 5.2. Adenosine and Fetal Programming

#### 5.2.1. Adenosine Metabolism and Function

**Figure 2:**
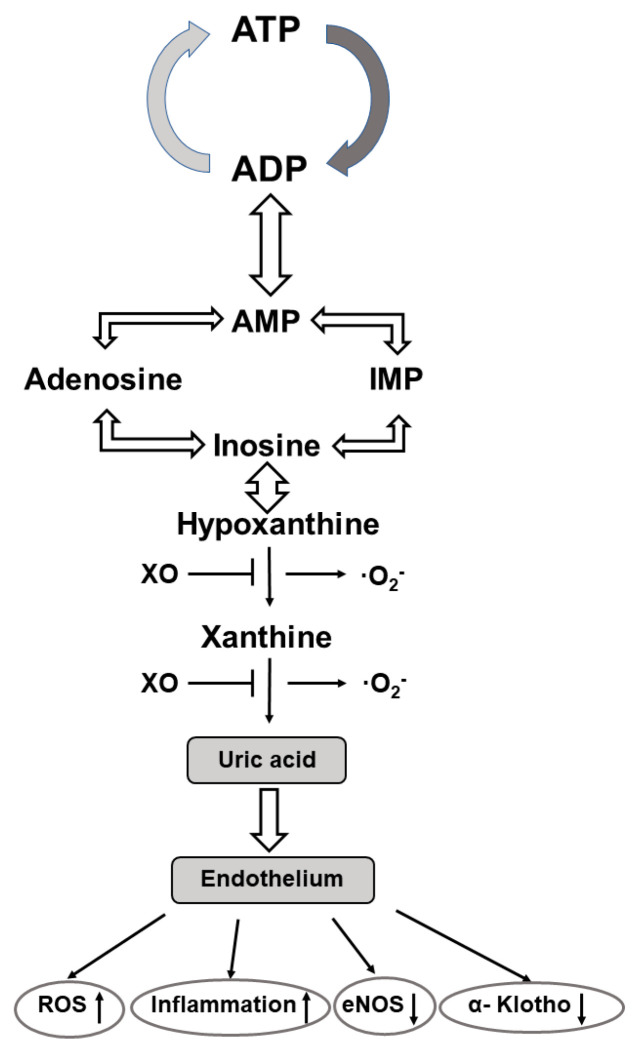
Metabolic pathway of the generation of uric acid and its effects on vascular endothelium. Abbre-viations: ATP—adenosine triphosphate; ADP—adenosine diphosphate; AMP—adenosine mono-phosphate; XO—xanthine oxidase; O_2_^−^—superoxide anion; ROS—reactive oxygen species; eNOS—endothelium nitric oxide synthase.

The purine nucleoside adenosine consists of adenine and the attached sugar molecule, ribose. It is generated through the degradation of adenosine tri/diphosphate (ATP/ADP) to adenosine monophosphate (AMP) via enzyme reactions of ectonucleotide triphosphate diphosphohydrolase 1 followed by dephosphorylation of AMP to adenosine via ecto-5′ nucleotidase [137,138]. It is involved in the regulation of multiple biological processes, including cardiovascular-, renal-, metabolic-, and immune-related functions [139]. Cell responses to adenosine are mediated through the activation of four distinct G-protein-coupled adenosine receptors (Ars) i.e., A1AR, A2AAR, A2BAR, and A3AR, respectively. These four Ars have distinct expression profiles, pharmacological characteristics, and signaling pathways [140]. Activation of AR1 and AR3 inhibits adenyl cyclase activity, cAMP production, and subsequent protein kinase A (PKA) activity, whereas activation of the AR2 subtypes stimulates adenyl cyclase, enhances the cAMP concentration, and finally results in an increased PKA activity. Furthermore, when A2B ARs are coupled to other G proteins they can stimulate inositol 1, 4, 5 triphosphate (IP3K) and diacylglycerol (DG) signalization [140]. All subtypes of ARs are expressed in the human fetoplacental endothelium, and they have been shown to have a regulatory role in angiogenesis, vascular tone, and placental blood flow [141] (Figure 2).

Under hypoxic conditions, ARs are differentially regulated in endothelial cells; in response to hypoxia mRNA for A2AAR is reduced, while mRNA for A2B is upregulated, respectively. Pharmacological manipulations with AR agonists and antagonists clearly revealed the proangiogenic function of all AR subtypes. It is of note, however, that A2A AR activation induces VEGF, IL10, and iNOS expression in macrophages, while activation of A2B AR increases VEGF, eNOS, and IL8 expression via the cAMP-PKA and the PI3K/AKT signaling pathways, respectively, in human microvascular endothelial cells. The angiogenic functions of A1AR and A3AR can be achieved without enhancing the expression/activity of iNOS/eNOS [142,143]. Importantly, adenosine has also been documented to increase cellular l-arginine uptake via the hCAT1 and l-arginine availability for NO generation. Based on the interplay between adenosine transport and the ARs with the l-arginine–NO system the concept of the adenosine/l-arginine/NO (ALANO) signaling pathway was developed and has been shown to operate in the human fetoplacental endothelium [143].

#### 5.2.2. Adenosine and Fetal Programming of Cardiometabolic Diseases

In a recent comprehensive review, Silva L and co-workers presented supporting evidence for the involvement of adenosine in the fetal programming of cardiometabolic diseases in offspring after their exposure to gestational diabetes mellitus [144]. In this regard, it could be considered that adenosine can also be derived from the folate-dependent methionine–homocysteine methylation cycle. SAM provides methyl groups via methyltransferases for methyl acceptors (including DNA, histones, and proteins, respectively) and is converted to SAH. Then, SAH is metabolized to homocysteine and adenosine by SAH hydrolase [145].

The major regulators of cellular adenosine levels are the enzymes adenosine kinase and adenosine deaminase, which catalyze the conversion of adenosine to AMP and to inosine, respectively. The reduced activity and expression of adenosine kinase results in increased cellular adenosine levels and angiogenesis, increased SAM and SAH levels, reduced activity of DNA methyltransferases, and alterations in the methylation-dependent gene expression. Moreover, adenosine kinase has been shown to modify the TNFα-induced gene expression profile of endothelial inflammatory markers in human umbilical vein endothelial cells (HUVECs) [144].

The pathogenetic role of adenosine signaling has also been documented in the development of preeclampsia. In a mouse model of preeclampsia induced by the selective deletion of placental adenosine deaminase, adenosine levels markedly and exclusively increased in the placenta. The pregnant mice with excess placental adenosine presented with major features of preeclampsia (including hypertension, and renal glomerular and placental vascular impairments) and with fetal growth restriction. These alterations could be prevented with adenosine deaminase therapy or by genetic deletion of the A2BAR, indicating that (a) the elevated placental adenosine could be attributed to its impaired elimination, (b) the effects of adenosine were mediated by A2BAR, and (c) adenosine deaminase was the key enzyme responsible for initiating the underlying biochemical changes of clinical preeclampsia and fetal programming [146].

### 5.3. Renin-Angiotensin System (RAS) and Fetal Programming

#### 5.3.1. Generation and Function of Angiotensin Peptides

**Figure 3:**
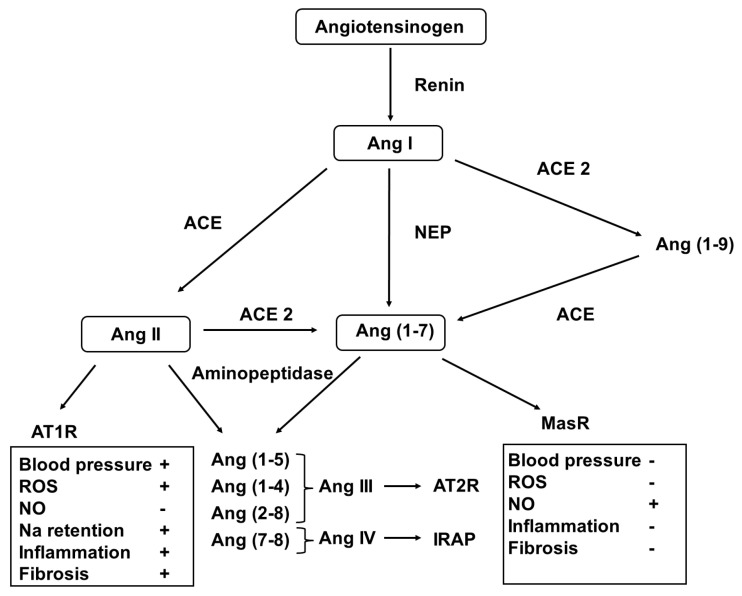
Metabolic pathway of the generation of angiotensin peptides. Abbreviations: Ang—angiotensin; NER—neprilysin; ACE—angiotensin-converting enzyme; ATR—angiotensin receptor; IRAP—insulin-regulated aminopeptidase; MasR—Mas receptor; ROS—reactive oxygen species; and NO—nitrogen monoxide.

In addition to the well-established role of the systemic renin-angiotensin-aldosterone system (RAAS) in maintaining blood pressure and volume and electrolyte homeostasis [147], it has been documented to act locally in an autocrine/paracrine fashion [148]. This notion has been supported with previous observations in that the elements of the RAS are expressed in many organs/tissues, including the kidney, brain, and cardiovascular and reproductive systems. Important studies have revealed the developmental pattern of the RAS, and it has been shown that targeted genetic and pharmacological interventions may disrupt its normal developmental course with the relevant clinical implications (Figure 3).

In the classical pathway, renin, released from the stored prorenin, catalyzes the formation of angiotensin I (AngI) from angiotensinogen. Then, it is converted to AngII by the angiotensin-converting enzymes (ACEs). AngII can be metabolized to form further bioactive peptides (AngIII2–8, AngIV3–6, and Ang1–7) and form the inactive Ang1–9. These conversions are achieved using ACE2 aminopeptidases and the membrane metalloendopeptidase [149].

Different Ang peptides act on distinct receptors (AT1R for AngII and AngIII, AT2R for AngIII), insulin regulated aminopeptidase (IRAP) for AngIV and MasR for Ang1–7. Activation of AT1R induces vasoconstriction, cellular hypertrophy and proliferation, fibrosis, and oxidative stress, as well as aldosterone and antidiuretic hormone release, that of AT2R and MasR results in vasodilatation, NO release, anti-hypertrophic, anti-proliferative, anti-fibrotic and anti-thrombotic effects. In addition, AngIV3–8 binding to IRAP stimulates nuclear factor kappa B (NFκB) and induces monocyte chemotactic protein-1, IL-6, intercellular adhesion protein-1 and plasminogen activator inhibitor-1 expression [150].

#### 5.3.2. RAS and Fetal Programming

The coordinated, well-balanced interplay of various ATR activations is essential for the functional integrity of RAS and for the subsequent normal development. In Ren-2 transgenic rats sodium-dependent hypertension develops, but the blockade of the ETA receptor decreases blood pressure and ameliorates end-organ damage [151]. ACE-deficient mice have a decreased blood pressure and severe renal impairments [152], while an ACE-2 deficiency itself causes hypertensive responses to AngII and dilated cardiomyopathy [153].

In humans, Ang-related genes are activated early in the developing embryo and are involved in the control of angiogenesis. ACE inhibitors in the first trimester of pregnancy were proven to be teratogenic and increased the risk of congenital malformations [154], while in mothers exposed to ACE inhibitors in the second and third trimesters the well-characterized fetopathy was deemed to potentially develop [155]. Similar fetotoxic effects have been reported after the maternal administration of angiotensin receptor blockers (ARBs) [156]. Based on these observations, the RAS dysfunction-related adverse fetal environment may increase the risk of renal and cardiovascular diseases later in life.

In a recent review by Yart L et al., important evidence has been provided for the critical role of the local utero-placental RAS in regulating the placental development and its functions. Namely, it has been shown to control implantation, trophoblast proliferation, angiogenesis, and utero-placental blood flow. Moreover, the involvement of the utero-placental RAS in modulating the placental endocrine and immune functions and nutrient transport has been established [157].

Under hypoxic conditions, AT1R expression is upregulated as a result of the increased production of pro-angiogenic factors required for placental vascular development. However, prolonged hypoxia over activates AT1R, and leads to the predominance of anti-angiogenic factors and reduction in the expression of the placental growth factor (PlGF), both contributing to impairments in placental perfusion. All these vascular changes are exclusively mediated by the trophoblastic AT1R signalization [157].

The function of the placental RAS has been extensively studied in pregnancies complicated with preeclampsia and fetal growth restriction. It has been clearly shown that the placental RAS is dysregulated with an imbalance between its vasoconstrictor and vasodilator components favoring to the AngII-AT1R-mediated pathological processes [158]. In addition, low aldosterone levels, along with accumulation of sodium by the negatively charged placental macromolecules cause volume depletion and further compromise placental perfusion [159].

AT1R-driven OS has also been identified as a major factor in the pathogenesis of vascular dysfunction in pre-eclamptic pregnant women and in the cardiovascular risk to their offspring [160]. In a series of experiments, Vaka R et al. demonstrated that administration of a selective AT1R agonist (AT1R-AAs-AT1 receptor agonist antibodies) to normal pregnant rats causes elevated mitochondrial ROS (mtROS) production in the placenta and the kidney. Furthermore, they also revealed that inhibition of AT1-AAs with the co-administration of a 1–7 amino acid peptide reduces mtROS in preeclamptic rats and have suggested that the AT1-AAs-mediated mitochondrial dysfunction may be achieved via the activation of NADPH oxidase. In addition, the anti-angiogenic factor sFlt-1, generated by the activation of AT1R, has also been claimed to stimulate placental and endothelial mtROS. However, this effect can be abrogated by the progesterone-induced blocking factor [161].

AngII-mediated endothelial dysfunction appears to persist postnatally. In preterm neonates, renal salt wasting, and sodium and volume depletion induces excessive RAAS activation to mitigate the sodium deficit and restore sodium levels [162]. Circulating ADMA, a marker of endothelial dysfunction, presents a similar postnatal increase and time course during the study period of five weeks. It is tempting to postulate, therefore, that the activated renin-angiotensin II—AT1R pathway, either directly or indirectly via the generation of superoxide, enhances the redox-sensitive ADMA accumulation [163]. It is also to be considered that the production of the vasoactive adrenal steroid ouabain is also elevated, and its elevation proved to be dependent of the activity of the RAAS [164]. As ouabain has been suggested to mediate superoxide and peroxynitrite production, it may also contribute to endothelial dysfunction, and to the development of cardiovascular diseases later in life [165,166].

## 6. Renal Development and Perinatal Programming

### 6.1. Nephrogenesis—Fetal Nephron Endowment

The fetal kidney develops from three successive mesodermal structures: the pronephros, the mesonephros, and the metanephros. The pronephric tubule extends into the mesoderm of the mesonephros to form the Wolffian duct. The mesonephros develops from an outbranch of the Wolffian duct termed the ureteric bud, which extends into the undifferentiated metanephric mesenchyme. Reciprocal inductive interactions between the ureteric bud epithelium and the metanephric mesenchyme result in the formation of the collecting duct system and the nephrons of the permanent kidney. The ureteric bud grows and branches repeatedly and forms the renal collecting system and induces mesenchymal transformation to form the glomeruli and renal tubules. These complex processes are under the control of metanephric mesenchyme-derived inductive signals (Wilms’ tumor gene1-WT1 and glial cell line-derived neurotrophic factor-GDNF) and transcription factors, including Pax2, Lim1 and the Formin gene [167].

Nephrogenesis begins at around 8 weeks of gestation and is completed by weeks 34–35. Afterwards, nephrons grow only in size. Nephrogenesis follows a centrifugal pattern: juxtamedullary nephrons mature more rapidly than those located superficially. The average nephron number per kidney is about 750,000 with wide inter-individual variations (250,000–1,500,000). The nephron number is inversely proportional to the glomerular volume. The variability in the nephron number was claimed to be due to genetic factors, the intrauterine environment, and maternal conditions. Low birthweight caused by either fetal growth restriction or preterm birth has been found to be associated with a reduction in the nephron number, and positive correlations have been established between the birthweight and the final nephron endowment. In addition to a low birthweight, nephrogenesis can be compromised by many other factors, including lifestyle, diet restriction, exposure to drugs (excess glucocorticoid, nephrotoxic antibiotics, and RAS inhibitors) maternal vitamin A and iron deficiency, and gestational diabetes. The adverse fetal environment may initiate adaptive responses at any states during nephrogenesis but may manifest only decades later as renal functional impairments and hypertension or may accelerate the progression of renal diseases. Early postnatal insults due to intensive care may also compromise renal development (second hit) and may contribute to the programming of renal diseases later in life [168].

### 6.2. Gestational Chronodisruption and Programming of Renal Dysfunction

More recently, the importance of gestational chronodisruption during the fetal programming of renal dysfunction and hypertension has been documented. In female pregnant rats exposed to chronic photoperiod shifting (CPS) throughout gestation, the renal effects of CPS were evaluated on the fetus and adult offspring. It was shown that in the kidney at the 18 day gestation timepoint, the clock- and clock-controlled gene expression did not present the daily circadian pattern. Furthermore, DNA microarray analysis has revealed differential expression for 1703 transcripts in CPS relative to the control fetal kidney. Alterations have also been observed in diverse gene networks of the CPS fetal kidney involved in transcription regulation, aldosterone-dependent Na reabsorption, and connective tissue differentiation.

In adult offspring of CPS mothers at 90 days of age, the circulating pro-inflammatory cytokines were elevated and different genes with specific renal functions (including genes for the glucocorticoid and mineralocorticoid receptors) were affected. Protein expression of kallikrein and COX-2 was reduced, and hypertension developed particularly when a diet high in NaCl was provided [169]. Importantly, maternal chronodisruption throughout pregnancy has been demonstrated to influence the developmental programming of glucose and adipose tissue homeostasis [170]. Melatonin has been proven to be a key hormone signal from the mother to the fetus, and maternal melatonin treatment was successfully applied to rescue the endocrine, inflammatory, and transcriptional influences of gestational chronodisruption in adult female rat offspring [171].

The significance of chronodisruption in the development of chronic kidney disease has been increasingly recognized, and disruptors have been identified to implement novel therapeutic approaches [172].

### 6.3. Renin-Angiotensin-Aldosterone System (RAAS) and Fetal Renal Programming

It has long been recognized that in addition to transcription factors, growth factors, adhesion molecules, and their receptors, the RAAS plays an important role in the control of renal morphogenesis and functional development [155,170,171,172,173,174,175,176,177,178,179,180]. In support of this notion, renin-expressing precursor cells and other elements required to generate bioactive AngII and its receptors, AT1R and AT2R, are expressed in the metanephros. Angiotensinogen and ATR2 genes are involved in controlling glomerular endothelial growth and the fetal glomerular hemodynamics [181,182,183]. Furthermore, the mutational inactivation of the AT2R gene has been identified to be associated with the CAKUT sequence (congenital anomalies of the kidney and urinary tract) [184]. The glomerular vascular effects of AngII in the developing kidney is mostly mediated by AT1R. However, AngII-induced vasoconstriction is counterbalanced by the activation on AT2R that is achieved either directly or indirectly by the stimulation of the vasodilator NO, bradykinin, and prostaglandins [185].

More recently, the potential role of the ACE2-Ang(1–7)–MasR-NO axis in the fetal/perinatal programming of the adult renal and cardiovascular diseases has been extensively studied. It has been shown that in response to fetal programming events (antenatal glucocorticoid exposure, low-protein diet in animal models, or preterm birth in humans) the activity of the protective ACE2-Ang(1–7)-MasR pathway is markedly reduced, and the classical ACE-AngII-AT1R pathway predominates that results in endothelial dysfunction with ROS generation, inflammation, sodium retention, blood pressure elevation, and tissue fibrosis [178]. Interestingly, the multiple developmental kidney injury induced by the genetic inactivation or pharmacological inhibition of ACE or AT1R cannot be abolished by the deletion of the ACE2 or MasR genes [181].

In human studies, neonates born preterm have lower plasma Ang(1–7) levels in the umbilical cord blood than their full-term counterparts, and these differences persist into adolescence and young adulthood. Namely, at the age of 14 years adolescents born preterm have reduced plasma Ang(1–7) levels and a higher AngII to Ang(1–7) ratio as compared with those born at term. This difference proved to be more pronounced in female adolescents and in individuals with obesity [178].

At the age of 14 and 19 years blood pressure is consistently elevated in preterm vs. term born adolescents and is associated with an increased ratio of plasma AngII to Ang(1–7). In adolescents born preterm, the lower urinary excretion of Ang(1–7) is associated with a reduced α-Klotho protein excretion and increased serum uric acid levels. It was suggested, therefore, that each may contribute to the perinatal programming of the later cardiovascular and renal diseases [178]. In this regard, it is to be considered that (a) the α-Klotho protein may confer protection to the vascular endothelium due to its anti-inflammatory, antioxidant, and anti-apoptotic properties [186], and (b) uric acid has also been documented to stimulate tissue RAS and AngII production that mediates oxidative stress, inflammation, endothelial dysfunction, and vascular smooth muscle cell proliferation. Additionally, ROS is also generated during purine base catabolism when xanthine oxidoreductase catalyzes the formation of hypoxanthine to xanthine, and xanthine to uric acid, respectively [187]. Applying xanthine oxidoreductase inhibitors is a promising way to limit uric acid and their related ROS production and attenuates the progression of biochemical processes before the clinical manifestation of the cardiovascular and renal diseases. With this contention in line, the role for xanthine oxidase inhibitors in the control of fetal cardiovascular function in late gestation sheep has been established. It was achieved by reducing fetal uric acid plasma levels and ROS generation, and by enhancing NO bioavailability and β1-adrenergic stimulation [188]. Preventive administration of allopurinol has also been proven to effectively alleviate post-hypoxic-ischemic reperfusion injuries, including fetal brain injuries [189,190].

### 6.4. Glomerular Hyperfiltration and Fetal Programming

**Figure 4:**
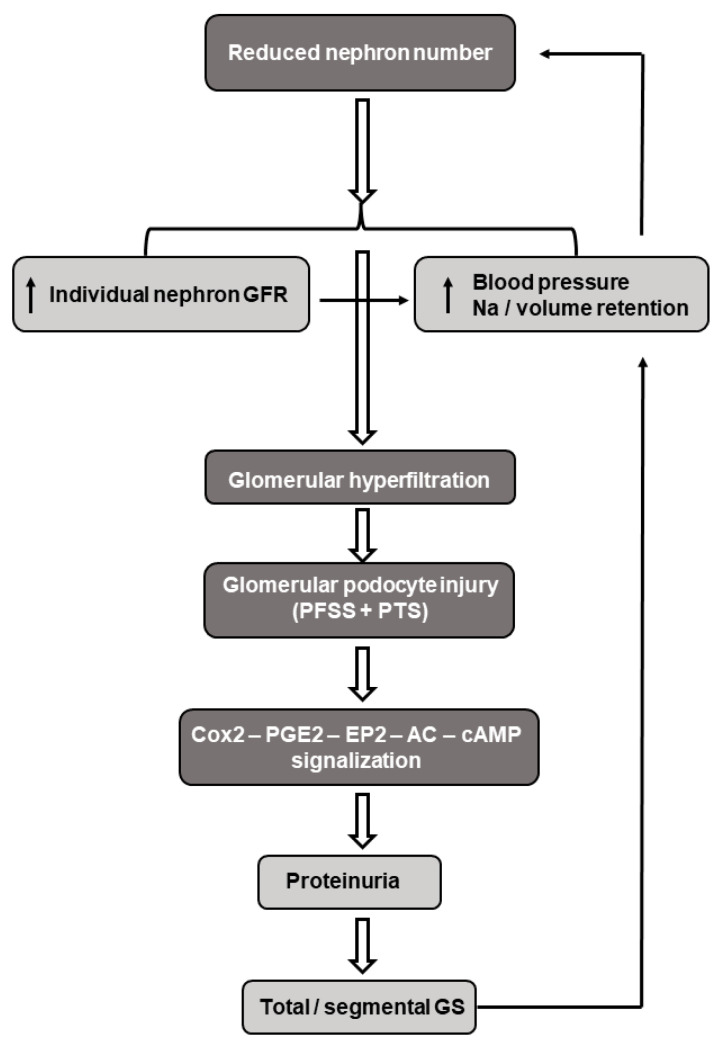
Schematic presentation of the pathomechanism(s) underlying the reduced nephron number induced by glomerular hyperfiltration, hypertension, and nephron change. Abbreviations: GFR—glomerular filtration rate; FFSS—fluid flow-induced shear stress; PTS—pressure-induced tensile stress; Cox2—cyclooxygenase 2; PGE2—prostaglandin E2; EP2—prostanoid receptor; cAMP—cyclic adenosine monophosphate; and GS—glomerular sclerosis.

Brenner et al. developed the concept in that the reduced nephron endowment at birth may serve as a risk factor for progressive renal injury and adult hypertension. A low nephron number leads to a decreased filtration surface area with a subsequent increase in the intra-glomerular pressure and hyperfiltration of the individual glomeruli. These hemodynamic changes may result in podocyte destruction, inflammatory mediator release, and fibrosis with subsequent proteinuria and focal/segmental glomerulosclerosis [191,192]. Furthermore, intraglomerular formation of AngII is increased and the pro-inflammatory arm of the local RAS (AngII-AT1R axis) is activated, which predominates over the anti-inflammatory, vasodilator ACE2-Ang(1–7)-MasR pathway. This imbalance may lead to the AngII-mediated vasoconstriction of the efferent arterioles, elevated intraglomerular pressure, and lead to the need to maintain hyperfiltration, that in the long-run may progress to hypertension and chronic kidney disease [193]. In this regard, it is to be underlined that the relationship between hypertension and chronic kidney disease is reciprocal; elevated blood pressure causes glomerular hyperfiltration-related renal impairments, which in turn increases the blood pressure. In support of this notion, markedly reduced nephron numbers have been detected in patients with hypertension [194] (Figure 4).

Increased renal tubular sodium reabsorption and the subsequent volume expansion have also been claimed to contribute to the developmental programming of hypertension and salt sensitivity. This notion has been supported by previous observations which indicated that the prenatal exposure to a low-protein diet or glucocorticoids in rats induced the upregulation of the bumetanide-sensitive Na-K-2Cl and thiazide-sensitive Na-Cl cotransporters [195], as well as the proximal tubular Na+/H+ antiporter [176] in adult offspring. It appears, however, that this concept does not apply to humans. Renal immaturity in low-birthweight, preterm neonates causes renal salt-wasting, high rates of fractional sodium excretion, markedly reduced Na+/H+ exchanges [196,197,198], and low expressions of the α-subunit of the epithelial Na channel (α-ENaC) mRNA [199]. Importantly, the developmental course of the α1-Na-K-ATP-ase mRNA runs parallel with that of the α-ENaC mRNA in various species [200,201]. To reconcile these apparently conflicting observations, one can assume that sodium depletion in the low-birthweight premature infants during the first weeks of life may initiate counter-regulatory reactions to restore their sodium balance. When these reactions persist, excess sodium may be retained with long-term cardiovascular and renal consequences.

The maladaptive reactions to a nephron deficit have been claimed to be the major underlying mechanism in the developmental programming of adult hypertension and renal diseases. However, the critical role of the nephron number in perinatal programming process has been questioned, and evidence has been provided in that the timing, type, intensity, and duration of intrauterine/early postnatal insults, along with the gender of the offspring also have to be considered [174].

This contention has been substantiated by Luyckx and Brenner, who have also proposed that the low nephron number at birth alone (except for the severe one) may not lead to kidney dysfunction, but when challenged by superimposed transient or permanent kidney injury, the kidney having fewer nephrons cannot cope efficiently with the functional demand and clinically apparent renal diseases may develop as a result. With this contention in line, they formulated the “multiple developmental hits” theory, implying that in addition to in utero insult(s) to the fetus, further insults during the life course may accelerate nephron loss, deterioration of renal function, and development of chronic kidney diseases. The first hit is caused by an adverse intrauterine environment. It may include prematurity, growth restriction, and large birthweights. Furthermore, maternal lifestyle factors, chronic illness, pregnancy pathologies (preeclampsia, gestational diabetes, maternal under/overweight, medications, exposure to maternal/fetal hypoxia, and glucocorticoids) are the major factors to be considered. The second hit is related to the neonatal intensive care, including imbalanced nutrition (calorie, protein, amino acids, and sodium intake), exposure to nephrotoxic drugs, and renal ischemia/reperfusion. During the life course, further hits (overt kidney diseases and conditions with acute kidney failure) may occur that accelerate the age-related progressive nephron loss and deteriorate renal functions. The multiple hits concept is of primary importance to underscore the complexity in the developmental programing of kidney diseases, and to timely implement the appropriate measures to preserve the renal functions [202].

## 7. Nutritional and Metabolic Fetal Programming

It has long been recognized that maternal nutrition acts as a critical risk factor for programming metabolic syndrome, diabetes, hypertension, cardiovascular diseases, and obesity occurring in offspring later in life. Based on epidemiological and experimental studies, Symonds et al. have established the nutritionally sensitive developmental windows in sheep and have emphasized that nutritional manipulations at these defined stages of gestation exhibit different impacts on embryonic/fetal growth and development and life-time health outcomes. Namely, maternal nutrient restriction during early gestation mostly affected the brain and cardiovascular functions as manifested as a smaller brain, impaired mental and behavioral capacity, and an altered carotid baroreflex that proceeds to later manifest as hypertension. Nutrient restriction during early-to-mid gestation reduced placental weight and its potential to inactivate maternal cortisol through 11 β-hydroxysteroid dehydrogenase type 2. Furthermore, dietary interventions at this stage impaired nephrogenesis along with the reduction in the nephron number. In this fetal phase of gestation, maternal undernutrition decreased the birthweight, fetal adipose tissue deposition, and muscle mass followed by an increased fat accretion, glucose intolerance, and insulin resistance at one year of age [14].

Fetal undernutrition and growth restriction has been claimed to be frequently associated with excess glucocorticoids and a reduced pancreatic β cell mass/secretory capacity that may lead to dyslipidemia, endothelial dysfunction, and glucose intolerance/insulin resistance predisposing to metabolic syndrome and/or type 2 diabetes mellitus in later life [203,204]. When prenatal nutrient restriction is followed by postnatal nutrient abundance, the risk of later life consequences are amplified. In addition to maternal/fetal undernutrition, mothers with a high nutrient intake, obesity, increased pregnancy weight gain, pre-pregnancy diabetes, or gestational diabetes are at risk of having large-for-dates neonates that are predisposed to have obesity and cardiometabolic diseases in adulthood. The developmental programming of offspring obesity has been shown to be achieved by the dysfunction of the hypothalamic appetite/satiety pathway and increased adipogenesis and lipogenesis as these processes are responsive to alterations in the nutrient environment [205].

The association of maternal obesity with vascular health has been extensively studied by Pardo et al. They have revealed that mothers with a normal pre-pregnancy weight but with supra-physiological gestational weight gains (spGWG) (total weight gain > 16 kg, its rates > 0.42 kg/weeks) have a reduced foeto-placental vascular reactivity due to the downregulation in eNOS expression and activity, as measured in the HUVECs and umbilical vein. This endothelial dysfunction has been assumed to be related to the altered adenosine transport. The impact of low-grade inflammation and OS has not been addressed, but a newborn phenotype of spGWG has been defined implying that its adverse cardiometabolic profile may predispose to programming adult diseases [206]. Extending these observations to gestational diabetes mellitus, they have reported increased activities in the l-arginine–NO pathway and NO-induced limited adenosine uptake in human placental vascular endothelial cells. These alterations could be recovered with insulin, confirming the potential role of the insulin/adenosine/placental endothelial dysfunction in pregnancies complicated with gestational diabetes mellitus [207].

### 7.1. Metabolic Hormones and Fetal Programming

#### 7.1.1. Insulin

Insulin is the key hormone in the metabolic adaptation to pregnancy. During the second half of pregnancy, insulin resistance develops with hyperinsulinemia, along with an increase in the β cell mass due to β cell hypertrophy and proliferation. This β cell reaction has been claimed to be accounted for by placental lactogen, prolactin, and growth hormone stimulation. Offspring exposed to maternal obesity, pre-pregnancy diabetes, gestational diabetes, or maternal over-nutrition during gestation and/or lactation are predisposed to be born macrosomic, and to develop obesity and glucose intolerance later in their adulthood. Fetuses of obese and/or overfed mothers may have low-grade inflammation, pancreatic fat deposition, and fibrosis resulting in β cell dysfunction with a diminished insulin secretion. Maternal undernutrition during gestation has been shown to reduce the birthweight and β cell mass at birth that may gradually progress to glucose intolerance, insulin resistance, and type 2 diabetes later in life [208].

The underlying mechanisms of a reduced β cell mass in fetal undernourished offspring are not clearly defined. The contribution of elevated corticosteroid levels in decreasing the number of Ngn3 endocrine progenitor cells, mitochondrial dysfunction, diminishing the tolerance to oxidative stress, and apoptosis have all been proposed [208]. Clinical and experimental studies have shown that insulin plays an important role in the control of endothelial function [189]. It operates in two opposite ways by activating two distinct (A and B) receptors. On the one hand, it is vasoprotective; stimulates endothelial NO generation through the phosphoinositide 3-kinase (PI3-K)-AKT-NOS pathway. On the other hand, it activates the mitogen-activated protein kinase (MAPK)-dependent signaling pathway, which regulates the vasoconstrictor ET-1. Under physiological conditions, these opposing endothelial effects of insulin are in balance. However, in pathologies associated with insulin resistance (oxidative and inflammatory stress and enhanced activity of the RAAS), insulin signaling is directed toward the MAPK-ET-1 pathway at the expense of the PI3-K-NO pathway. This imbalance may lead to endothelial dysfunction that may lead to the remodeling of the vascular wall. In the foeto-placental vasculature both insulin receptors A and B are expressed, and the related metabolic pathways operate. It is relevant to assume, therefore, that in pregnancy pathologies with insulin resistance the insulin receptor B-MAPK-ET-1 pathway predominates and compromises the foeto-placental circulation [209,210,211].

#### 7.1.2. Irisin

Irisin has also been documented to play a role in fetal metabolic dysregulation and the long-term programming of obesity and metabolic syndrome. Irisin is an exercise-induced myokine consisting of 212 amino acids that is cleaved from its transmembrane protein precursor, fibronectin type III domain-containing protein 5 [212]. Its major source is the skeletal muscle, but several other tissues, including the placenta, have also been identified to secrete irisin. In the umbilical cord, blood plasma irisin was markedly reduced in growth-restricted neonates and correlated positively with the birthweight and plasma insulin levels [213]. As irisin functions to promote the browning of the white adipose tissue, to improve insulin sensitivity in insulin-resistant states, and to protect functional integrity of the vascular endothelium, its deficiency in the fetoplacental circulation may therefore amplify the susceptibility of growth-retarded neonates to the later development of metabolic syndrome. The positive correlation between irisin and insulin in large-for-dates neonates has been interpreted as a protective irisin reaction to mitigate insulin resistance and excessive fat deposition [214,215,216].

#### 7.1.3. Leptin

Leptin, a 16-kD protein hormone is encoded by the obese gene and is produced by adipocytes in proportion of the body fat mass [217]. It functions as a metabolic signal from the adipose tissue to brain areas regulating food intake, energy storage, and energy expenditure. Its actions are mediated through specific leptin receptors by inhibiting neuropeptide-Y-secreting neurons in the hypothalamus. As leptin secretion is influenced by several hormones, it is regarded as a part of complex metabolic and neuroendocrine regulatory system [218,219,220]. Its clinical significance in the perinatal period, and its possible involvement in the metabolic programming of adult diseases are being explored. In our early studies, it was found that maternal serum leptin levels steadily increase during the period of 12 to 28 weeks of gestation without any further changes until term. These changes in the maternal serum leptin appear to follow the patterns of the gestational alterations in the maternal fat tissue mass [221].

Evidence has also been provided for the neonatal characteristics of leptin metabolism. Namely, it has been found that (a) umbilical cord blood levels are markedly elevated without discernible differences in the arterial and venous cord sera of full-term neonates, (b) their leptin levels are closely related to the birthweight and to the rate of intrauterine growth, (c) there is a dramatic fall in leptin levels during the early postnatal life compared to their cord blood values, and that (d) boys have significantly lower cord blood leptin levels than girls. Moreover, premature infants with a mean gestational age of 30.2 weeks exhibited leptin levels that were significantly lower than their mature counterparts. During the first five postnatal weeks serum leptin levels in preterm male neonates increased progressively with weight gain but were inversely related to testosterone [222]. In a recent study of weight discordant twins, it has been shown that growth-retarded twins demonstrate insulin resistance with decreased fetal and increased placental leptin levels as compared with co-twins exhibiting appropriate fetal growth. Concomitantly, soluble leptin receptor levels increased in growth-restricted twins limiting leptin access to the membrane-bound receptor, and thus further enhancing leptin resistance [223]. Fetal leptin derives from the placenta with a less significant contribution in the fetal adipose tissue. This contention is supported by the observations in that leptin mRNA is expressed in the placental tissue (syncytiotrophoblast-facing maternal circulation; villous vascular endothelium-facing fetal circulation), placental weight is significantly related to the cord blood leptin levels, and there is an abrupt fall in the leptin levels postpartum [224,225,226]. Considering the multiple functions of leptin derived either from the placenta or from the fetal/neonatal adipocytes and its greatly elevated level during the perinatal period, one can assume that defective leptin signalization may contribute to the development of metabolic disorders in later life.

#### 7.1.4. Adiponectin

Adiponectin is a protein hormone consisting of 218 amino acids. It is mainly expressed in, white adipose tissue and has insulin-sensitizing, anti-inflammatory, anti-atherogenic, and anti-proliferative properties. In human circulation, three forms of adiponectin have been identified: the low, -middle- and high-molecular weight forms. The high-molecular weight form is the most abundant in the plasma, and its reduction has been associated with metabolic disorders (obesity, metabolic syndrome, and type 2 diabetes) [227,228,229]. As reviewed by Arroyo-Jousse et al., in human pregnancy adiponectin decreases late in pregnancy in parallel with its reduced mRNA expression in the white adipose tissue; however, the human placenta does not express adiponectin. The biological actions of adiponectin are achieved by activating its two receptors: AdipoR1 and AdipoR2, respectively. Both receptors are expressed in term placentas, but only AdipoR2 activation stimulates the p38 MAPK and PPAR-α signaling pathways and inhibits glucose and amino acid transport. Adiponectin has also been shown to enhance apoptosis and to promote the migration and invasion of trophoblast cells in the first trimester placenta. Women with pre-gestational obesity and overweight pregnancy have lower adiponectin levels along with a lower expression of placental adipokine receptor genes than in women with a normal weight. Maternal adiponectin levels are positively related to those of their offspring and to the rate of fetal growth at the third trimester of the patients and are inversely related to the birthweight and subcutaneous fat in the neonate. Cord blood adiponectin is associated with body fat mass at birth and later in early childhood [230].

The role of adiponectin in controlling placental functions and fetal growth has been underscored by the observations in that adiponectin deficiency of adiponectin knockout or heterozygote pregnant mice increased the expression of genes involved in placental fatty acid transport and caused triglyceride accumulation in the fetal liver. Wildtype dams on a high-fat/high-sucrose diet protected their fetuses from fatty acid overload [231]. Accordingly, adiponectin supplementation in obese pregnant mice prevented the adverse effects of maternal obesity on placental function and fetal growth. Furthermore, it normalized insulin sensitivity, placental insulin/mechanistic target of rapamycin complex 1 (mTORC1) and PPARα signaling, nutrient transport, and fetal growth [232]. Importantly, Bouchard et al. have described the adaptive increases of circulating adiponectin in response to maternal hyperglycemia and insulin resistance. This reaction was associated with the hypomethylation of the adiponectin gene DNA in human placenta, indicating that epigenetic mechanisms are involved in maintaining maternal/fetal glucose homeostasis [233].

## 8. Conclusions and Perspectives

Since the discovery of the fetal origin of certain chronic diseases later in life, great progress has been made in our understanding of the pathomechanism(s) underlying these complex processes. It has been established that environmental insults/stressful stimuli (maternal lifestyle, pre-pregnancy diseases, pregnancy pathologies, impaired placental functions, disturbed postnatal adaptation, neonatal intensive care, formula feeding, catch-up growth, and toxic substances) can induce adaptive responses that allow a single phenotype to manifest as different phenotypes of the offspring. These new phenotypes can be transmitted to the next generation, or even to further generations. Developmental programming is confined to a relatively short period (“a critical window”) when developmental plasticity prevails. This period allows us to implement therapeutic interventions to prevent or to re-program developmentally programmed adult diseases.

In this narrative review, we provide an outline of the major pathogenetic factors involved in the process of developmental programming. These include OS, inflammation, telomere dysfunction, and epigenetic modifications via DNA methylation, histone acetylation, and microRNAs. Hypoxic insults can activate inflammatory cells and enhance the production of inflammatory mediators culminating in the fetal systemic inflammatory response syndrome. Inflammation in turn, leads to an increased ROS release, resulting in a vicious circle to ensue. Moreover, important data are presented on the role of the RAS/RAAS, l-arginine–NO–ADMA system, purine catabolism, and reduced nephron endowment of the maturing kidney, as well as of the nutrition and major metabolic hormones.

Concerning the complexity of the pathomechanisms of the developmental programming and the coordinated interplays between these distinct pathogenetic factors, it is a challenge to timely introduce targeted preventive/therapeutic approaches. Instead, general measures, including lifestyle interventions, avoidance of maternal/fetal over/under nutrition, improved pregnancy care, less invasive neonatal intensive care, and an extended period of breast feeding should all be provided. In addition, diet balanced in micro- and macronutrients, antioxidant administration, supplementation with methyl group donor molecules (folate, methionine, choline, and betaine) and with 3-n polyunsaturated fatty acids have been proven to be beneficial.

It appears that differential DNA methylation, enhancement of protective protein expression (α-Klotho, SIRTs, and erythropoietin) and pharmacological restoration of the delicate balance between the classical RAS and the protective ACE 2- Ang(1–7)-MasR system may have the potential to reduce the risk of the developmental programming of chronic diseases in later life.

## Data Availability

Not available.

## Abbreviations

OS	Oxidative stress
TERT	Telomerase reverse transcriptase
TERC	RNA template of telomerase reverse
HIF-1α	Hypoxia-inducible factor 1α
IUGR	Intrauterine growth restriction
ROS	Reactive oxygen species
CpG	Cytosine bases preceding guanine
DNMT	DNA methyltransferase
SAM	S-adenosylmethionine
SAH	S-adenosylhomocysteine
HAT	Histone acetyltransferase
HDAC	Histone deacetylase
TET	Ten-eleven-translocation
5-hmC	5-hydroxy-methylcytosine
5-mec	5-methylcytosine
Sirtuin	SIRT
NO	Nitrogen oxide
eNOS	Endothelial nitric oxide synthase
VEGF	Vascular endothelial growth factor
CAT	Cationic amino acid transporter
nNOS	Neuronal nitric oxide synthase
iNOS	Inducible nitric oxide synthase
ADMA	Asymmetric dimethylarginine
MMA	Monomethylarginine
PRMT	Protein methyltransferase
DDAH	Dimethylarginine dimethylamino hydrolase
cGMP	Cyclic guanosine monophosphate
ATP	Adenosine triphosphate
ADP	Adenosine diphosphate
AMP	Adenosine monophosphate
AR	Adenosine receptor
cAMP	Cyclic adenosine monophosphate
PKA	Phosphokinase A
IP3K	Inositide-1,4,5 triphosphate kinase
PI3K	Phosphatidylinositol 3-kinase
DG	Diacylglycerol
IL-10, IL-8	Interleukin-10, -8
AKT	Protein kinase B
TNFα	Tumor necrosis factor α
HUVEC	Human umbilical cell
RAAS	Renin-angiotensin-aldosterone system
RAS	Renin-angiotensin system
Ang	Angiotensin
ACE	Angiotensin-converting enzyme
MasR	Angiotensin—(1–7) receptor, class A G protein-coupled receptor
NFκB	Nuclear factor kappa B
ET	Endothelin
ARB	Angiotensin receptor blocker
PLGF	Placental growth factor
AT1R-AAS	Angiotensin 1 receptor agonist antibodies
mtROS	Mitochondrial reactive oxygen species
